# Proactive and reactive inhibitory control are differently affected by video game addiction: An event‐related potential study

**DOI:** 10.1002/brb3.2584

**Published:** 2022-04-25

**Authors:** Mazyar Fathi, Shahrzad Mazhari, Ali Mohammad Pourrahimi, Ahmad Poormohammad, Sara Sardari

**Affiliations:** ^1^ Neuroscience Research Center Institute of Neuropharmacology Kerman University of Medical Sciences Kerman Iran; ^2^ Parsian Hearing and Balance Center, Shahin Shar Isfahan Iran; ^3^ Department of Psychiatry Medical School Kerman University of Medical Sciences Kerman Iran

**Keywords:** ERP, proactive inhibition, reactive inhibition, stop signal task, video game addiction

## Abstract

**Introduction:**

Video game addiction (VGA) is associated with physical and mental disorders, one of which is problem in executive function, particularly inhibitory control. The present study aimed to investigate reactive and proactive inhibitory controls by event‐related potential (ERP).

**Methods:**

Thirty video game (action video games)‐addicted subjects and 30 matched healthy controls participated in the study, who were tested by the selective stop‐signal task.

**Results:**

The main results revealed that the VGA group had significantly more problems in preparatory processes and proactive stop trials, showing that VGA has a negative effect on proactive inhibition.

**Conclusion:**

Finding the problem in proactive inhibitory control might be helpful in developing new treatments and rehabilitation methods in these fields.

## 1 INTRODUCTION

Many people play video games for entertainment, but excessive video gaming can lead to video game addiction (VGA) (A. Weinstein et al., 2017). The World Health Organization included gaming disorder as a mental health disorder in the 11th revision of the International Classification of Diseases. Excessive video gaming causes adverse effects on mental and physical health and can lead to auditory hallucinations, anxiety, wrist and neck pain, and seizure (A. Weinstein et al.,, 2010). Moreover, studies have demonstrated that individuals with VGA show impairment in some cognitive functions such as executive function, inhibition, attention, working memory, and decision‐making (Kuss et al., 2018; Raud & Huster, 2017).

Response inhibition is defined as an active process involved in cancelling a planned movement (Aron, 2007; Muller & Anokhin, 2012; Nigg et al., 2006). It is an essential factor for self‐regulation, adaptation, and controlling behavior in different situations (Aron, 2007; Muller & Anokhin, 2012; Wu et al., 2010). It has been suggested that problem in response inhibition is a predictive marker for behavioral and drug addictions (Bartholdy et al., 2016; Nigg et al., 2006; F. Verbruggen et al., 2013). Response inhibition consists of two main components: reactive and proactive inhibitions (Aron, 2011). Recent studies have well demonstrated that for a successive response inhibition, both reactive and proactive inhibitions are required (Braver, 2012; Jaffard et al., 2008). In reactive inhibition, subjects stop the response outright when commanded by signal, while in proactive inhibition, the inhibitory mechanism has already started and subjects are prepared to stop the upcoming behavior (Aron, 2011). Moreover, proactive inhibition is developed according to the goals of the subject and triggered by both endogenous and exogenous factors, but the reactive inhibition is only triggered by signal and exogenous factors (Braver, 2012; Jaffard et al., 2008). In addition, in contrast to reactive inhibition, proactive inhibition is goal‐directed and needs to be mediated by working memory to handle the information about how and when inhibition should be implemented (Meyer & Bucci, 2016).

Many studies have evaluated the response inhibition in gaming disorder by simple Go/No‐Go task and have reported problems in conflict monitoring and inhibitory control in terms of N2 and P3 and the impulsivity effect (Dong et al., 2010; Littel et al., 2012; Zhou, Yuan, Yao, Li, & Cheng, 2010). However, no study has explored the different types of inhibitory control (proactive and reactive), which may contribute to VGA.

Selective stop‐signal task has been used to study proactive and reactive inhibitions. In this task, probability of the stop signal can be predicted by presenting a cue in each trial. When there is a possibility of a stop signal, the person's strategy changes to proactive to produce a correct response and this slows down the reaction time in Go trials. In addition, selective inhibition is studied by the modified task, in which the Go stimulus requires a bimanual speeded response and the stop signal appears only for one hand, while the other hand's response is still expected to be executed (Raud & Huster, 2017).

Event‐related potential (ERP) with a high temporal resolution have been suggested as a sensitive method to study response activation and response inhibition. P3 Cue correlates to the attentional preparatory processes and reflects the attentional resources needed for the correct response to the expected target (Doehnert et al., 2010; Knight, Grabowecky, & Scabini, 1995; Spronk et al., 2008), and assessment of the stimulus (Grane et al., 2016; Karayanidis et al., 2009). P3 Go is related to the source of attention, updating the information related to the task, and response selection (Overtoom et al., 1998; Spronk et al., 2008). N2 stop is related to conflict monitoring and interference processing (Raud & Huster, 2017). P3 stop is suggested to be an online indicator of response inhibition and related to evaluation of the inhibitory process (Huster et al., 2011; Raud & Huster, 2017).

This study aimed to investigate both proactive and reactive inhibitions by selective stop‐signal task in individuals with VGA who play action video games. The results of this study may provide new insights for any clinical therapy that targets motor inhibition.

## 2 METHODS

### 2.1 Participants

A total of 30 subjects with VGA were chosen from two local game clubs. The following requirements were used to determine eligibility: male gender, being right‐handed, aged 17 to 35 years, playing action video games, playing 30 h or more online video games per week for at least 1 year, and a VGA test (VAT) evaluation score of 2.5 or higher (Luijten, Meerkerk, Franken, van de Wetering, & Schoenmakers, 2015; van Rooij, Schoenmakers, van den Eijnden, Vermulst, & van de Mheen, 2012). The control group consisted of 30 male participants who were of similar age and had a VAT score of 1.5 or less (van Rooij et al., 2012). Drugs or alcohol abuse (with the exception of cigarette smoking), traumatic brain injury, mental and neurological disorders, psychotropic medication use, and a history of memory disorders were all exclusion criteria for both groups. They were asked not to smoke for 1 h before the experiment to eliminate the effect of nicotine on brain output.

Participants were asked to fill out four self‐reported questionnaires. (1) To determine game addiction, one VAT was used. It consisted of 14 self‐reported questions on a four‐point Likert scale ranging from 0 to 4 (never to very often) (Luijten et al., 2015). The possible scores ranged from 0 to 5, with higher scores suggesting greater severity of the condition. (2) Barratt Impulsiveness Scale 11 (BIS‐11) was used to assess impulsivity (Patton , & Barratt, 1995). (3) Beck Depression Inventory (BDI) (Becket al., 1996), and (4) Beck Anxiety Inventory (BAI) (Beck et al, 1988) were used to assess depression, and anxiety, respectively. Handedness was determined based on the Edinburg inventory (Oldfield, 1971). All of the participants signed a written informed consent. Kerman University of Medical Sciences' ethics committee approved the study (Ethics code: IR.KMU.REC.1397.279).

### 2.2 Procedure assessment

Subjects sat in a sound‐attenuated dimly lit room that met the ANSI S3.1‐1999 standard during EEG recording. After completing the SST tasks, they filled all the questionnaires (VAT, BIS, BDI, and BAI). In a relaxed posture, the participants sat in a comfortable chair with their heads fixed on a chin rest. The participants were 1.5 m away from a 17‐inch monitor screen. Psytask software, version 1.53.17 was used to design the task (Mitsar Inc., Russia).

### 2.3 Experimental task

**FIGURE 1:**
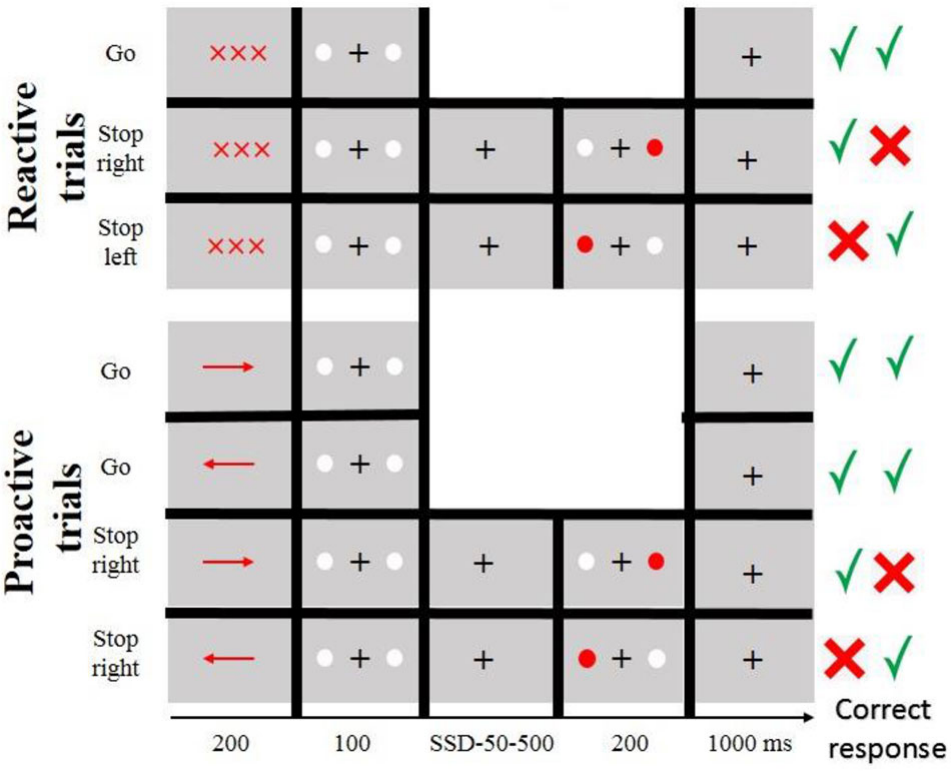
Selective stop signal task in the two condition of reactive and proactive, performed by informative cues in proactive and noninformative cues in reactive conditions, all trails presented in pseudorandom order

In total, 700 trials were presented, 350 of which were reactive and the other half were proactive. Trials in the reactive condition began with a noninformative cue with 200 ms duration, while trials in the proactive condition began with informative arrows pointing to left or right. They were followed by a 1300 ms gap filled with a fixation cross in the screen's center. Then, in the Go trials (60 percent of all trials), go stimuli were presented for 100 ms by two white circles with a diameter of 2.5 cm and a distance of 10 cm between them, during which the participants had to push two buttons at the same time. In the stop trials (40 percent of all trials), a red circle appeared at the left or right location that was previously occupied by the go stimuli, and the respondent was required to suppress the hand to the side of the red circle. This red circle with a duration of 200 ms appeared after a stop signal delay (SSD) that was randomly modified, with a duration ranging from 50 to 500 ms. Because all cues were congruent, informative ones in stop trials could help participants start preparing for a potential stop (proactive stop trials). There were 420 Go trials and 140 stop trials in each condition, with 70 trials at the left and 70 trials at the right, while stop trials were split into two directions, left and right, Figure 1 (Raud & Huster, 2017).

In this study, omission error in the Go trial was defined as not responding to Go trials. Go‐RT was defined as the elapsed time between the onset of a Go trial and response to it, and was considered for both reactive and proactive trials. Choice errors in unsuccessful stop trials: unsuccessful stop trials in which an incorrect Go response was executed (stimulus required a left response but a right response was executed). This parameter was considered for both reactive and proactive trials in left and right directions. Stop signal reaction time (SSRT) was defined as latency of the stop process and evaluated by the integration method for reactive and proactive trials in both directions (Raud & Huster, 2017; F. Verbruggen et al., 2019). The integral method estimates the end of the stop process by “integrating” the RT distribution and finding the point where the integral equals p (response | signal). The stop processes finishing time related to the nth RT, n = distribution of a number of Go trial reaction times that multiplied by the probability of responding on stop trials in the same session and mean SSD was subtracted to this parameter for estimating SSRT(Eagle et al., 2008; F. Verbruggen et al., 2019).

### 2.4 ERP acquisition

A 32‐channel Win EEG system was used to record and evaluate EEG (version 2.126.97, Mitsar Inc.). The sampling rate was 500 Hz, and electrodes were placed according to the 10–20 system placement, with an electrooculogram electrode being placed below the left eye to detect eye movement noise. Electrode impedances were kept below 5 k, and low and high pass filters were set (0.1–45 Hz). EEG was computed using Win EEG software and recorded in a monopolar montage, with input signals referenced to the linked ear.

ERPs were computed for Cue, Go, and Stop trials. Artifact correction was performed in the following order: (1) the raw EEG was visually probed for high‐amplitude and high‐frequency noises, and noisy trials (more than 100 μv) were removed; (2) eye blink artifacts were corrected by zeroing the activation curves corresponding to eye blinks; (3) independent component analysis (ICA) was run to detect the components associated with eye movement and muscular noise (Sardari et al., 2019).

2.5 Component selection

ERPs’ data were analyzed after artifact rejection. “P3 cue” was defined as the maximum peak between 300 and 550 ms after presentation of the Cue stimulus over central electrodes, and P3 in Go and stop trials were determined as the largest amplitudes in latencies between 280 and 500 ms after presentation of the Go and Stop stimuli at central electrodes. N2 of stop trials were determined as the second negative peaks and time delay duration between 200 and 350 ms after presentation of the stop stimulus over frontal electrodes. ERPs were analyzed for frontal and central electrodes (Fz, F3, F4, Cz, C3, and C4) (Domnguez‐Centeno et al., 2018; Takayose et al., 2016)

## 3 STATISTICAL ANALYSIS

The independent samples *t*‐test was used to compare VGA and control group demographics, psychometrics, and SST performance parameters (reaction time, omission errors of Go trials, SSRT [right and left], and choice error on unsuccessful stop trials). A mixed effects repeated measures analysis of variance (ANOVA) was conducted for each ERP with two within‐subject factors: (i) trial type (Go, NoGo, and irrelevant trials) and, (ii) electrode locations (Fz, F3, F4, Cz, C3, and C4). Significant main effects, interactions, and follow‐up pairwise comparisons were examined after adjustment for multiple testing (Bonferroni). All the analyses were conducted using the Statistical Package for the Social Sciences software, version 20.

## 4 RESULTS

### 4.1 Demographic and clinical characteristics

The demographic and clinical characteristics of the study participants are presented in Table 1. The results showed that the two groups had no considerable difference in terms of age and education. There were no significant differences between the two groups for scores of Edinburgh, BDI, BAI, and BIS (all *p* > .05). The VGA group had significantly higher scores in VAT (*p* < .0001), and spent significantly more time (*p* = .002) and days (*p* < .0001) on playing video games.

**TABLE 1:** Demographic and clinical characteristics of the study participants (mean ± SD)

**Variable**	**VGA**	**Controls**	***p* value**
Age (year)	20.39 ± 3.03	19.91 ± 1.94	.45
Edinburgh	78.19 ± 14.84	75.74 ± 22.28	.59
BDI	16.00 ± 11.64	16.44+10.95	.87
BAI	15.42 ± 12.12	15.45 ± 12.05	.99
BIS‐11	65.26 ± 11.30	64.59 ± 7.51	.78
VAT	2.82 ± .28	1.05 ± .62	<.000^∗^^∗∗^
Day/Week	6.50 ± .77	2.75 ± 2.12	<.000^∗∗∗^
Hours/Day	5.72 ± 1.24	1.34 ± 1.11	<.000^∗∗∗^

*Note*. BAI, Beck Anxiety Inventory; BDI, Beck's Depression Inventory; BIS11, Barratt Impulsiveness Scale–11; VAT, Video game Addiction Test; DAY/WEEK, Number days per week spent gaming, HOURS/DAY, Number of hours per day spent gaming. (∗: p≤ 0.05. ∗∗: p≤ 0.01. ∗∗∗: p≤0.001)

### 4.2 Behavioral data

Table 2 shows the behavioral data of the SST of the two groups. Regarding reaction time in the Go trials, the results showed that the VGA group had significantly faster RT in both reactive (*p =* .009) and proactive (*p =* .011) trials than the control group. In addition, in proactive stop trials, SSRT‐right differed significantly between the two groups and the VGA group had longer SSRT compared to the control group (*p* = .013), while no significant difference was observed between the two groups in reactive SSRTs. Regarding error rate, in proactive stop trials, the VGA group had significantly more choice errors in the stop right (*p =* .007) and stop left trials (*p =* .049). In the Go trials, the omission error was not significantly different between the two groups.

**TABLE 2:** Behavioral results on the SST for VGA patients and control groups

	VGA	Control		VGA	Control	
	Reactive			Proactive		
	mean ± SD	mean ± SD	*p* value	mean ± SD	mean ± SD	*p* value
SSRT‐RIGHT	343.2 ± 46.55	345.25 ± 33.31	.879	380.48 ± 48.40	339.75 ± 49.40	.013*
SSRT‐LEFT	357.08 ± 55.30	358 ± 38.38	.954	342.44 ± 45.02	334.50 ± 44.89	.58
CHE‐ STOPR	4.57 ± 3.319	3.13 ± 1.746	.113	5.70 ± 3.27	3.06 ± 2.32	.007*
CHE STOPL	5.03 ± 2.65	3.94 ± 2.23	.167	4.93 ± 3.60	2.94 ± 2.14	.049*
OM‐Go	20.72 ± 24.34	25 ± 14.34	.52	22.44 ± 24.95	22.94 ± 12.96	.942
RT‐Go	650.60 ± 10.70	735.13 ± 67.31	.009*	655.68 ± 101.89	729.88 ± 56.27	.011*

*Notes*, SSRT, stop signal reaction time; CHE‐STOPR, choice errors in stop right trials; CHE‐STOPL, choice errors in stop left trials; OM‐Go, number of omission errors in Go trials, RT, reaction time.(∗: p≤0.05. ∗∗: p≤ 0.01. ∗∗∗: p≤0.005)

Group means and standard deviations (SDs) are reported.

### 4.3 Electrophysiological data

**FIGURE 2:**
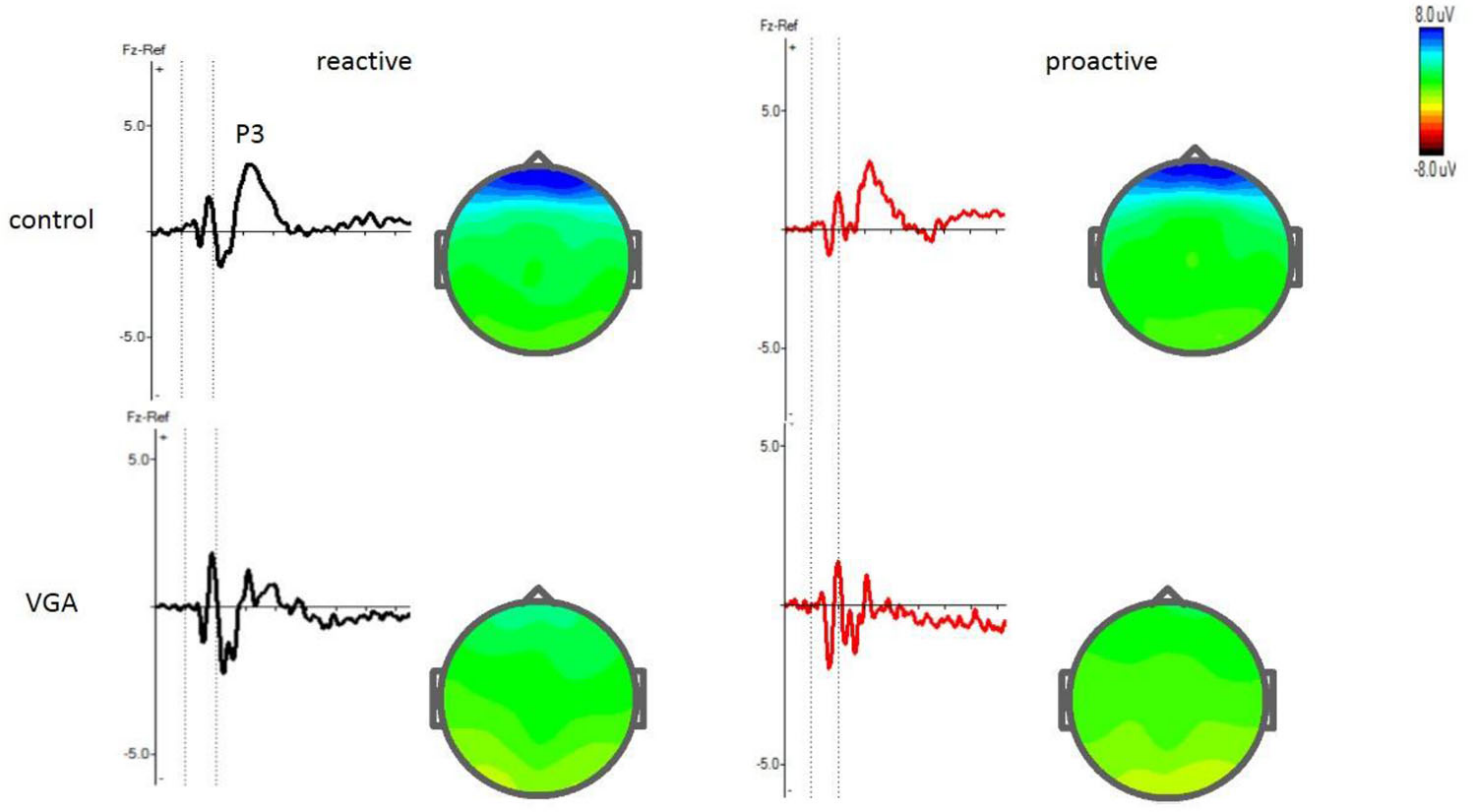
Grand average ERP waves over frontal electrodes for the VGA and the control subjects in Cue stimulus in both reactive and proactive conditions in the selective stop‐signal task and shows p3 component at Fz, electrode. The vertical dotted lines show the duration of stimulus. Topographic maps of the grand average peak amplitudes at Fz for P3‐reactive and P3‐proactive also shown

**FIGURE 3:**
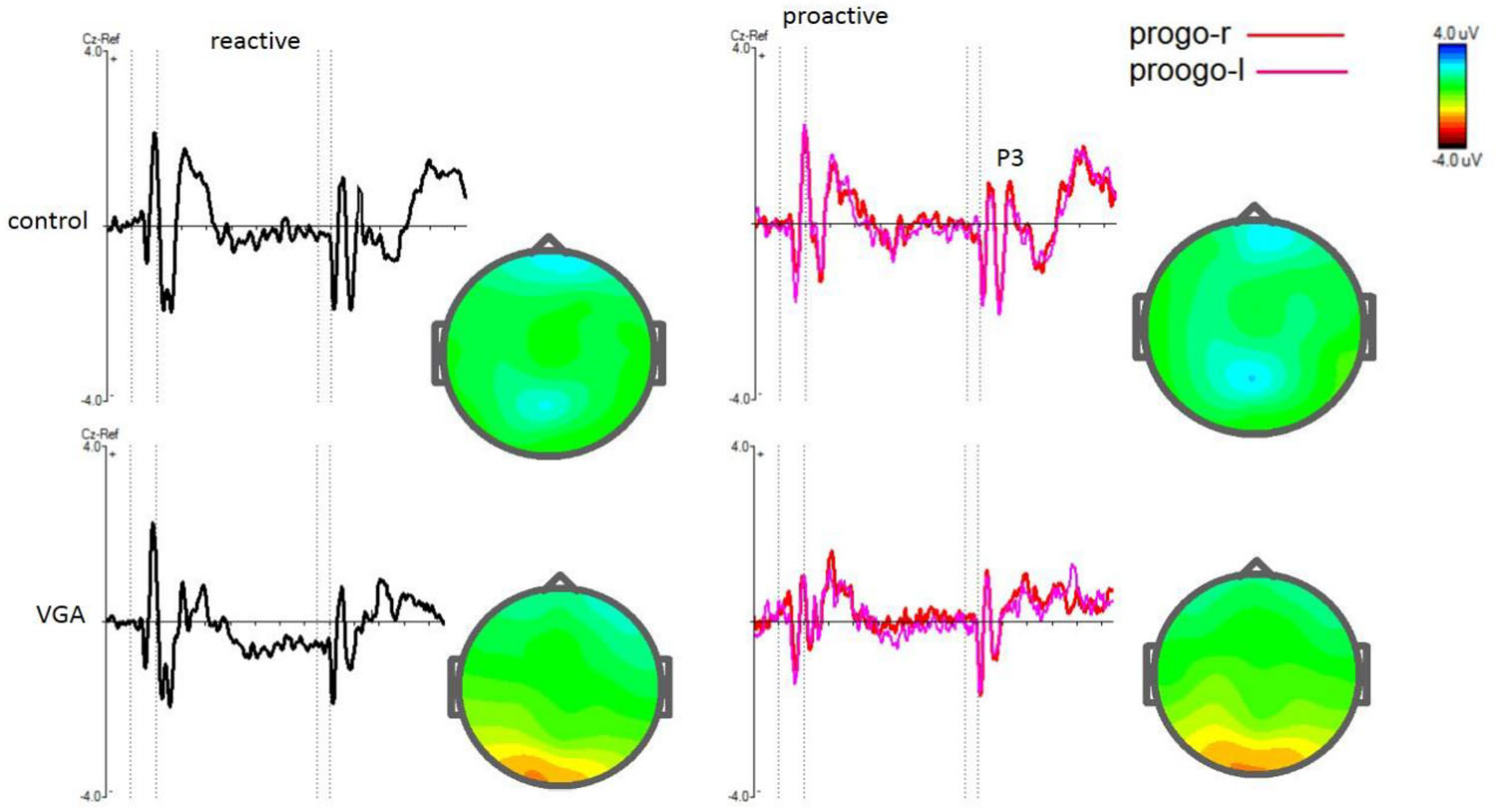
Grand average ERP waves over frontal electrodes for the VGA and the control subjects in Go stimulus in both reactive and proactive(right and left) conditions in the selective stop‐signal task and shows p3 component at Cz, electrode. The vertical dotted lines show the duration of stimulus. Topographic maps of the grand average peak amplitudes at cz for P3‐reactive and P3‐proactive also shown. Progo‐r: proactive go right, progoleft: proactive go left

**FIGURE 4:**
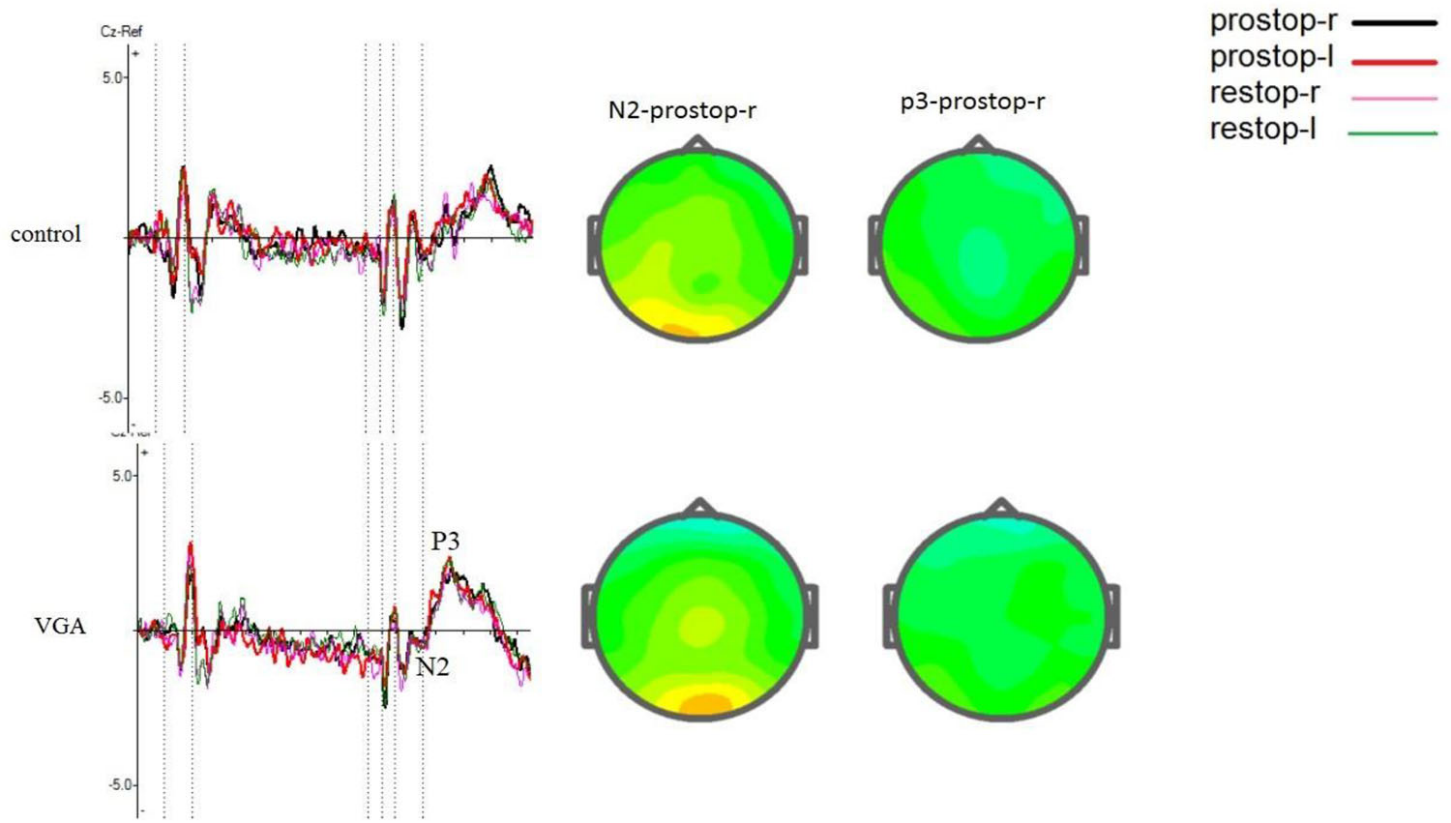
Grand average ERP waves over central electrodes for the VGA and the control subjects in stop stimulus at both reactive (right‐left) and proactive (right and left) conditions in the selective stop‐signal task and shows N2 and p3 components at Cz, electrode. The vertical dotted lines show the duration of stimulus. Topographic maps of the grand average peak amplitudes at cz for P3‐proactive trials also shown. Prostop‐r: proactive stop right, prostop‐l: proactive stop left, restop‐r: reactive stop right, restop‐l: reactive stop left

Table 3 shows mean amplitude of P3 for Cue and Go trials and N2 and P3 for stop trials in both groups. The grand averages of N2 and P3 for each group are presented in Figures 2, 3, 4.

**TABLE 3:** Mean ERP amplitudes (μV) for VGA patients and control groups

	VGA	Control		VGA	Control	
	Reactive			Proactive		
	mean ± SD	mean ± SD	*p* value	mean ± SD	mean ± SD	*p* value
**P3 cue**						
Fz	2.32 ± 1.04	3.95 ± 1.73	<.000	2.03 ± 1.36	3.3827 ± 1.76	<.000^∗∗∗^
F3	2.20 ± .84	4.2926 ± 1.90	<.000	5.43 ± 17.77	4.31 ± 8.512	.613
F4	2.36 ± .91	4.02 ± 1.72	<.000	2.12 ± 1.162	3.66 ± 1.682	<.000^∗∗∗^
**P3 Go**						
Cz	2.39541.66826	1.92 ± 2.11		2.19 ± 1.75	2.34 ± 2.11	
C3	1.94 ± 1.39	2.33 ± 1.57		1.82 ± 1.38	2.50 ± 1.67	
C4	2.24 ± 1.40	2. ± 1.83		2.02 ± 1.52	2.40 ± 1.64	
**N2 Stop‐right**						
Fz	‐.64 ± .53	‐.94 ± .570		‐1.2341.80538	‐2.8022 ± 1.17	<.000^∗∗∗^
F3	‐.61 ± .50	‐.42 ± .658		‐.99 ± .703	‐1.96 ± 1.12	<.000^∗^^∗∗^
F4	‐.44 ± .37	‐.45 ± .64		‐1.1190.76906	‐2.15 ± 1.03	<.000^∗∗∗^
**N2 Stop‐left**						
Fz	‐.45 ± .46	‐1.02 ± .62		‐1.37 ± .86	‐1.86 ± 1.07	
F3	‐.64 ± .53	‐.67 ± .48		‐1.1248.68873	‐1.10 ± .80	
F4	‐.44 ± .55	‐.62 ± .47		‐1.38 ± .88	‐1.43 ± .97	
**P3 Stop‐right**						
Cz	3.43 ± 1.73	3.21 ± 2.20		4.33 ± 2.74	2.83 ± 2.02	<.000^∗∗∗^
C3	2.84 ± 1.81	3.29 ± 2.64		3.75 ± 2.34	2.98 ± 1.56	.025^∗^
C4	3.07 ± 2.24	3.06 ± 2.05		3.58 ± 2.14	2.64 ± 1.70	.006^∗∗^
**P3 Stop‐left**						
Cz	3.7 ± 1.98	3.04 ± 2.89		4.33 ± 3.11	3.91 ± 2.52	
C3	3.03 ± 1.83	2.63 ± 3.20		3.46 ± 2.45	3.45 ± 2.81	
C4	3.22 ± 1.84	3.02 ± 2.54		3.45 ± 2.62	3.42 ± 2.46	

Group means and standard deviations (SDs) are reported.(∗: p≤ 0.05. ∗∗: p≤0.001. ∗∗∗: p≤0.001)

#### 4.3.1 ERPs to cue signals

Repeated measure ANOVA showed that there was a significant main effect of group on P3 amplitude [F (1, 140 = 7.83), *p* = .004, ŋ2 = 0.053]. Follow‐up analyses demonstrated that the VGA group exhibited significantly smaller amplitudes for both reactive and proactive cue stimuli (*p* > .05). There was no significant main effect of trial on the P3 amplitude [F (1, 140 = 0.595), *p* = .449, ŋ2 = 0.053], indicating that the P3 amplitude did not change in different trials. The results showed that there was no significant main effect of group in terms of P3 latency [F (1, 58 = 0.058), *p* = .81, ŋ2 = 0.001].

#### 4.3.2 ERPs to Go trials

The analyses showed that there were no significant main effects of group [F (1, 53 = 0.177), *p* = .676, ŋ2 = 0.003] and trial on P3 amplitude [F (1, 53 = 0.1), *p* = .753, ŋ2 = 0.002]. In term of latency, there was a significant main effect of group on P3 latency of Go trials [F (1, 54 = 7.50), *p* = .008, ŋ2 = 0.122] and post‐hoc test revealed that the control group had shorter latency compared to the VGA group in reactive and proactive trials (*p* < .05).

#### 4.3.3 ERPs to stop trials (N2)

In reactive stop trials, the analyses showed that there was no significant main effect of group [F (1, 60 = 1.47), *p* = .23, ŋ2 = 0.024] and no significant effect of stop direction (Stop right or Stop left) on N2 amplitude. Similarly, there were no main effects of group [F (1, 56 = 1.94), *p* = .169, ŋ2 = 0.032] and stop direction [F (1, 56 = 0.324), *p* = .571, ŋ2 = 0.006] on N2 latency.

In proactive stop trials, results showed that there were significant main effects of group [F (1, 60 = 11.324), *p* = .001, ŋ2 = 0.156], stop direction [F (1, 60 = 19.18), *p* < .000, ŋ2 = 0.242], and interaction of group with stop direction [F (1, 60 = 45.932), *p* < .000, ŋ2 = 0.434] on N2 amplitude. Follow‐up analyses indicated that the VGA group had significantly smaller N2 amplitude than the control group in stop right trials (*p* < .000), but there was no significant difference in stop left trials. This data did not show any significant main effect of group [F (1, 60 = 3.27), *p* = .076, ŋ2 = 0.052] and direction [F (1, 60 = 1.356), *p* = .391, ŋ2 = 0.012] on N2 latency.

#### 4.3.4 ERPs to stop trials (P3)

In reactive stop trials, there were no main effects of group [F (1, 139 = 0.454), *p* = .502, ŋ2 = 0.003] and stop direction [F (1, 139 = 0.021), *p* = .886, ŋ2 = 0.002] on P3 amplitude. In P3 latency, there were significant main effects of group on P3 latency in the stop‐right trials [F (1, 55 = 5.643), *p* = .021, ŋ2 = 0.093]; and the comparison analysis showed that in the stop right, the control group had shorter latency than the VGA group, but there was no significant main effect of direction on P3 latency [F (1, 55 = 3.318), *p* = .074, ŋ2 = 0.057].

The analysis of proactive stop trials showed a significant main effect of group on P3 amplitude [F (1, 133 = 4.66), *p* = .033, ŋ2 = 0.034] and follow‐up analysis indicated that P3 amplitude in the stop right was larger in the VGA group compared with the control group, while there was no significant main effect of direction on P3 amplitude [F (1, 133 = 1.27), *p* = .262, ŋ2 = 0.009]. In terms of latency, there were no significant main effects of group [F (1, 51 = 2.391), *p* = .128, ŋ2 = 0.045] and direction [F (1, 51 = 0.26), *p* = .872, ŋ2 = 0.001] on P3 latency in the stop right.

## 5 DISCUSSION

The purpose of this study was to evaluate the proactive and reactive inhibitory controls in VGA subjects. The main results showed that subjects with VGA had faster reaction time in both reactive and proactive Go trials, and they only had more choice errors and longer SSRTs in proactive trials. Moreover, analysis of ERPs demonstrated that the P3 amplitude of cue was larger in the control group in comparison with the VGA group in both reactive and proactive conditions, while the VGA group had smaller N2 amplitude and larger P3 amplitude, only in the stop right of the proactive trials. Additionally, in the Go trials, the control group showed shorter latency in P3 compared with the VGA group in both reactive and proactive trials.

### 5.1 Performance in the Stop‐signal task

At the behavioral level, the results demonstrated that the VGA group had more choice errors and longer SSRTs in proactive stop trials but not in reactive stop trials. Similarly, individuals with gambling disorder have also shown some difficulties in proactive inhibition (F‐Verbruggen et al., 2012). The findings of the current work indicate that excessive video gaming might affect the time needed to stop processing in cognitive and motor levels. In addition, more choice errors in stop trials may show problems in inhibitory control (F. Verbruggen et al., 2019).

Consistent with previous studies, the results showed that the VGA group had faster RTs in both reactive and proactive conditions (Bialystok, 2006; Castel, & Drummond, 2005). However, other studies have shown no significant differences between VGA group and controls (Kim et al., 2017). The discrepancies of the results may be related to various types of games with different effects on cognitive control (Boot & Simons, 2011; Nelson & Strachan, 2009). There are two insights about faster reaction time in video gamers: (i) experience of a video game decreases reaction time (Dye, & Bavelier, 2009), and (ii) people with faster RT are more intent to play (Latham & Tippett, 2013). Whenever a quick response is prioritized, it may decrease accuracy in response (Aasen & Brunner, 2016).

### 5.2 Electrophysiological performance in the selective stop‐signal task

The ERP results demonstrated that the VGA group had smaller P3 amplitude of cue than the control group in both proactive and reactive conditions. It should be noted that previous studies have proposed that P3 cue is related to allocation of attentional resources to expected targets (Doehnert et al., 2010; Knight et al., 1995), evaluation of stimulus, and activation of correct response (Karayanidis et al., 2009). It has been proven that accurate performance in tasks depends on accurate representation of context information and maintaining the data in the delay between the cue and the target (Dias, & Javitt, 2003). Information about the probability of a stop‐signal and accurate interpretation of the cue modulate the inhibitory process (Grane et al., 2016). Therefore, the current findings indicate that VGA subjects may have difficulty in relevant cue interpretation and the preparatory process for response inhibition.

In this study, the VGA group showed more prolonged P3 latency compared with the control group in Go trials. Similar to these findings, (Kim et al., 2017) showed that individuals with internet gaming disorder had prolonged P3 latency in the Go/NoGo task (Kim et al., 2017), and suggested that P3 latency is related to the depth of stimulus processing and speed of cognitive processing (Fahaueret al., 2015; Salisbury et al., 2004). Together, these results could indicate the negative effects of VGA on cognitive processing in the execution stage.

In the stop trials, the VGA group exhibited decreased N2 amplitude in comparison to the controls in the right proactive stop trials. This finding is in line with previous studies that reported reduced N2 amplitude in people with VGA in NoGo trials of Go/NoGo task (Dong et al., 2010; Gros, Debue et al., 2020). The current results suggest difficulties in early stages of inhibition and conflict monitoring in VGA individuals, because it is thought that the N2 amplitude is a marker of inhibitory control in the early stages of inhibition (Bokura et al., 2001). Indeed, response suppression in healthy individuals results in greater N2 amplitude than the Go response, implying that N2 is related to early stages of response inhibition (Kim et al., 2017; Thomaset al., 2014). Moreover, previous studies have found that the anterior cingulate and medial orbitofrontal cortex are the sources of N2 generation in stop trials, and these cortical areas are involved in conflict monitoring and inhibitory control (Bokura et al., 2001; Jodo & Kayama, 1992). Interestingly, imaging studies have shown the negative effects of VGA in the function of these areas (Bailey et al., 2010).

In this study, reduced N2 amplitude was only observed in the proactive stop trials and not in the reactive ones in people with VGA. In agreement with this finding, (Bailey et al. 2010), found attenuated Medial Frontal Negativity wave in excessive gamers, which is related to proactive cognitive control, but no significant differences were observed in ERP components of reactive cognitive control (Bailey et al., 2010). Moreover, fMRI studies have found reductions in the functions of brain areas involved in proactive cognitive control, anterior cingulate, and lateral frontal cortex in excessive video gamers (Bailey et al., 2010). Overall, it could be concluded that people with VGA have problems in early stages of proactive inhibition that are confirmed by electrophysiological and behavioral results, while reactive inhibition is less affected in this group.

In contrast to reduced N2 amplitude, the VGA group exhibited increased P3 amplitude in the right proactive stop trials. Previous studies have shown different results in terms of increase and decrease in P3 amplitude in NoGo trials (Kim et al., 2017; Kuss et al., 2018), which may be related to differences in task difficulty or type of games. P3 stop reflects the mechanisms involved in withholding a prepotent motor response (Dong et al., 2010). The increase of P3 amplitude in this trial may be indicative of compensatory mechanisms that prevent premature response, or poor impulse control in VGA subjects.

In addition, electrophysiological analysis showed more differences in the right stop (smaller N2 and lager P3) between the groups, which were also confirmed by behavioral results. A previous study on healthy subjects indicated that right‐handed people have more errors and prolonged inhibitory process RTs in their right hand when a left‐hand movement is required, suggesting that their right hand is preselected to undertake tasks (Buckingham & Carey, 2015). Additionally, this phenomenon may be worse in VGA subjects, because they must have very fast reactions by their right hand when playing action video games.

In general, the main results of the present work revealed that the VGA subjects had smaller P3 cue amplitude, reflecting a weaker interpretation and preparatory process for producing a response. Moreover, they had smaller N2 and larger P3 in the proactive trials, which show problems in the early stages of the inhibitory process in these trials.

These findings have clinical implications, because impairments in inhibitory control have been linked to development of drug and behavioral addictions (Verbruggen et al., 2012), and recovery from addiction requires the inhibition of the addictive behavior (Verbruggen et al., 2012). Finding a link between VGA and impaired proactive inhibitory control suggests new avenues for clinical therapy aimed at motor inhibition.

## 6 LIMITATIONS AND FUTURE DIRECTION

This study had a limitation as only male subjects were selected and female participants should be included in future studies. Major problems were found in the stop‐right trials, thus, future studies are needed to reveal the underlying mechanisms of the issue regarding selective inhibition in the right hand. The finding of this research showed more commission errors and faster RT, which in future researches can be studied in terms of speed‐accuracy trade‐off strategy.

## CONFLICT OF INTEREST

The authors declare no conflict of interest.

### PEER REVIEW

The peer review history for this article is available at https://publons.com/publon/10.1002/brb3.2584

## Data Availability

The data that support the findings of this study are available on request from the corresponding author. The data are not publicly available due to [restrictions e.g. their containing information that could compromise the privacy of research participants].
